# Septal curvature as a robust and reproducible marker for basal septal hypertrophy

**DOI:** 10.1097/HJH.0000000000002813

**Published:** 2021-07-01

**Authors:** Maciej Marciniak, Andrew Gilbert, Filip Loncaric, Joao Filipe Fernandes, Bart Bijnens, Marta Sitges, Andrew King, Fatima Crispi, Pablo Lamata

**Affiliations:** a School of Biomedical Engineering and Imaging Sciences, Kings College London, London, UK; b Cardiovascular Ultrasound, GE Vingmed, Oslo, Norway; c Institute of Biomedical Research August Pi Sunyer (IDIBAPS), Barcelona; d Catalan Institution for Research and Advanced Studies (ICREA), Barcelona; e Hospital Clinic de Barcelona; f Barcelona Center for Maternal Fetal and Neonatal Medicine, Hospital Sant Joan de Déu, Barcelona, Spain

**Keywords:** basic research, curvature, diastolic function, hypertension, hypertrophy, systolic function

## Abstract

**Background:**

Basal septal hypertrophy (BSH) is an asymmetric, localized thickening of the upper interventricular septum and constitutes a marker of an early remodelling in patients with hypertension. This morphological trait has been extensively researched because of its prevalence in hypertension, yet its clinical and prognostic value for individual patients remains undetermined. One of the reasons is the lack of a reliable and reproducible metric to quantify the presence and the extent of BSH. This article proposes the use of the curvature of the left ventricular endocardium as a robust feature for BSH characterization, and as an objective criterion to quantify current subjective ‘visual assessment’ of the presence of sigmoidal septum. The proposed marker, called average septal curvature, is defined as the inverse of the radius adjacent to each point of the endocardial contour along the basal and mid inferoseptal segments of the left ventricle.

**Method:**

Robustness and reproducibility were assessed on a cohort of 220 patients, including 161 hypertensive patients (32 with BSH) and 59 healthy controls.

**Results:**

The results show that compared with the conventionally used wall thickness metrics, the new marker is more reproducible (relative standard deviation of errors of 7 vs. 13%, and 8 vs. 38% for intra-observer and interobserver variability, respectively) and better correlates to the functional parameters related to BSH, with main difference (absolute rank correlation 0.417 vs. 0.341) in local deformation changes assessed by longitudinal strain.

**Conclusion:**

Average septal curvature is a more precisely defined and reproducible metric than thickness ratios, it can be fully automated, and better infers the functional remodelling related to hypertension.

## Introduction

The increase in cardiac wall thickness in hypertensive heart disease is gradual and not uniform [1]. In early stages of hypertension, some patients demonstrate localized hypertrophy of the basal septum. The significance of this finding is not definitive; however, some studies indicate that it serves as a marker of a more advanced impact of afterload on cardiac function in patients with hypertension [2]. If a patient presents with localized thickening, there is reasonable concern that they have hypertension influencing the cardiac function and might benefit from more stringent blood pressure control and a detailed follow-up.

The link between this localized thickening and elevated blood pressure was demonstrated when volunteers with basal septal hypertrophy (BSH) and no known history of hypertension were diagnosed with masked hypertension using 24-h blood pressure monitoring [3]. In the general population, the Framingham Heart study found the prevalence of BSH to be at 1.5%, reaching up to 17.8% in older patients [4]. A number of other studies investigated the prevalence of BSH in hypertensive cohorts, finding it to be around 20% [5,6].

This morphological trait is thus an early sign of structural and functional remodelling [7], and has been extensively researched, in spite of a lack of consensus on nomenclature, with the finding being termed septal bulge [1,8,9], isolated interventricular septum thickening [5,10], discreet upper septal thickening [4,11,12], and basal septal hypertrophy [2,13,14], among others. Beyond the nomenclature, different criteria exist to define BSH, the majority of which are based on the myocardial thickness in the basal interventricular septum (IVS), and its relation to the thickness at the mid-point of the septum or the deviation from a normal value. For example, the basal septal wall thickness [10–13], basal septal and mid-septal wall thickness ratios (WTR) [1,5], various ratios of the septal thickness and other echocardiography parameters [9,12], or a combination of the above-mentioned [4,15] have all been used to define BSH. The reproducibility of these criteria is often limited as arbitrary cut-off values are employed by different groups (given the lack of gold standard), and the fact that the measurement of the thickness often relies on the M-mode. Moreover, the discrepancies in the metrics may occur for several reasons: inconsistency in finding the epicardial border, IVS measurements not exactly perpendicular to the septal wall and/or different opinions on the positioning of the basal and mid segments among the experts.

Apart from the quantitative wall thickness measurements, an equally important diagnostic criterion is the visual assessment of the septal geometry as the shape of the BSH is also distinctive. This diagnostic marker was termed a sigmoidal septum or an upper septal ‘knuckle’ [2,4,8,15,16]. The visual determination of the BSH is based on its location in the basal segment of the IVS, the fact that it is asymmetric and the visible myocardial thickening is more abrupt and convex than in the cases of concentric LV hypertrophy. Given the lack of clear guidelines, this assessment is subjective, and suffers from low reproducibility.

The problem addressed in this article is this lack of a clear criterion and reproducibility in the assessment of BSH. Together with the variability in the characteristics of the examined cohorts, this problem causes current difficulties in determining the prevalence and the associated risks of BSH. The solution proposed to distinguish between patients with and without BSH in a robust and reproducible manner is a combination of a semiautomatic segmentation of the left ventricle (LV) and the computation of curvature along the LV endocardium of the septal wall. The rationale is that curvature is a metric that objectively quantifies the subjective visual assessment of the presence of the sigmoidal septum, and thus removes human variability. The motivation is also based on the hypothesis that the curvature of the endocardial contour is more reproducible than metrics relying on highly variable thickness measurements.

A retrospective study of hypertensive patients is presented here in order to evaluate the performance of the septal curvature compared with the traditional methods. We analyse the intra-observer and inter-observer variability of the metrics and validate the hypothesis of the improved reproducibility of curvature. We then investigate the relationship of the anatomical BSH metrics with early markers of functional remodelling associated with hypertension, finding that curvature is a better descriptor of the deformation changes. We finally explore the relationship between thickness and curvature, both by their correlation and by the diagnostic agreement based on defined thresholds, and discuss the clinical relevance of our findings.

## Methods

Hypertensive patients (*N =* 161) with well-controlled blood pressure, treated with antihypertensive drugs for a minimum of 3 years, were included. Patients were recruited from the outpatient clinic and general practitioner referrals. Exclusion criteria included history of heart failure or previously known target organ damage. Volunteers from the local community (N = 59) who were presumed healthy, without prior history of hypertension, diabetes or other significant cardiac or noncardiac diseases represented healthy controls. Full description of the cohorts is included in the supplementary material, http://links.lww.com/HJH/B583.

Examinations were performed by qualified sonogra-phers according to the current recommendations of the European Association of Cardiovascular Imaging [17] on a commercially available GE Vivid 9 system equipped with a M5S transthoracic transducer. Machine settings, including gain, time gain compensation, and compression, were adjusted for optimal visualization. Images were analysed using GE EchoPAC software (v.202.41.0, GE Vingmed, Horten, Norway).

BSH is defined based on the basal-to-mid septal thickness ratio of at least 1.4 in either the four-chamber (4CH) or parasternal long-axis (PLAX) view [7]. In order to classify the cohort, thicknesses in the anteroseptum and inferoseptum were manually measured during end-diastole at basal-level and mid-level in PLAX and 4CH views, respectively. The measurements were obtained perpendicular to the LV long axis, at the interface between the myocardial wall and cavity to the transition of the LV to the RV septal myocardium [17]. On the basis of these criteria, 32 (20%) patients were diagnosed with BSH.

**Figure 1:**
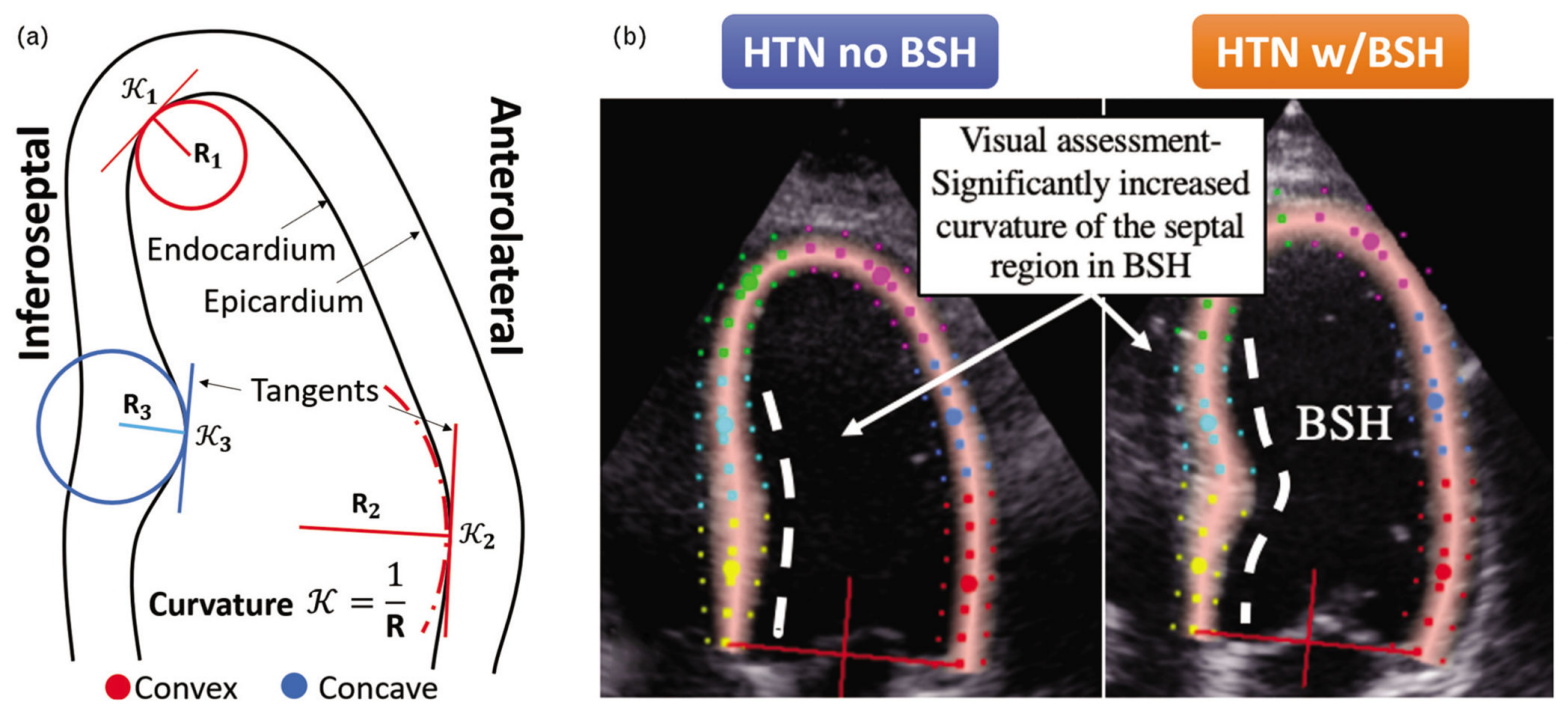
(a) An intuitive explanation of convex and concave curvature of the left ventricular endocardium, as seen in the four-chamber echocardiography view. The curvature is a reciprocal of the radius of circle adjacent to the endocardium. (b) The comparison of the septal wall in hypertensive patients with and without the basal septal hypertrophy (BSH). The visual assessment differentiating the two can be quantitatively compared with the curvature metric.

Curvature is the amount by which a line deviates from being straight; therefore, it is expected to be highest around the apex, and low at the mid and basal wall segments. Along a contour, three consecutive points in space define a circumference, and the smaller the radius of this circumference, the larger the curvature. With these intuitive ideas, the LV endocardial curvature is defined as the inverse of the radius of the circle that can be defined by triplets of adjacent points of the contour delineated on the endocardium. We consider it being concave if the adjacent circle has its centre outside of the left ventricular cavity and convex otherwise (see Fig. 1a). The curvature in the LV septum provides information about the convexity of the hypertrophy directly quantifying the visual assessment performed by clinicians (Fig. 1b).

In practice, curvature of a 2D curve is calculated with its first and second derivatives and is quantified with inverse length units (dm^−1^) [18]. Let *c(t)* = *(x(t),y(t))* be a representation of a curve with existing, continuous first and second derivatives, defined in 2D space. For each point *t* of the trace defined on the 2D plane, the curvature *κ* is calculated as (Eq. 1):

$$\kappa (t)=\frac{x{\left(t\right)}^{\prime \prime }y{\left(t\right)}^{\prime }-x{\left(t\right)}^{\prime }y{\left(t\right)}^{\prime \prime }}{{\left(x{\left(t\right)}^{\prime 2}+y{\left(t\right)}^{\prime 2}\right)}^{3/2}}$$

where *x(t)′*, *x(t)″* are the first and second derivatives in point *t* along one of the spatial directions.

Although the curvature value must always be positive [i.e. the absolute value of (Eq. 1)], the LV endocardial contour may contain both convex and concave curvatures, and to distinguish between the two, we consider the curvature convex if the calculated value is positive and concave otherwise.

**Figure 2:**
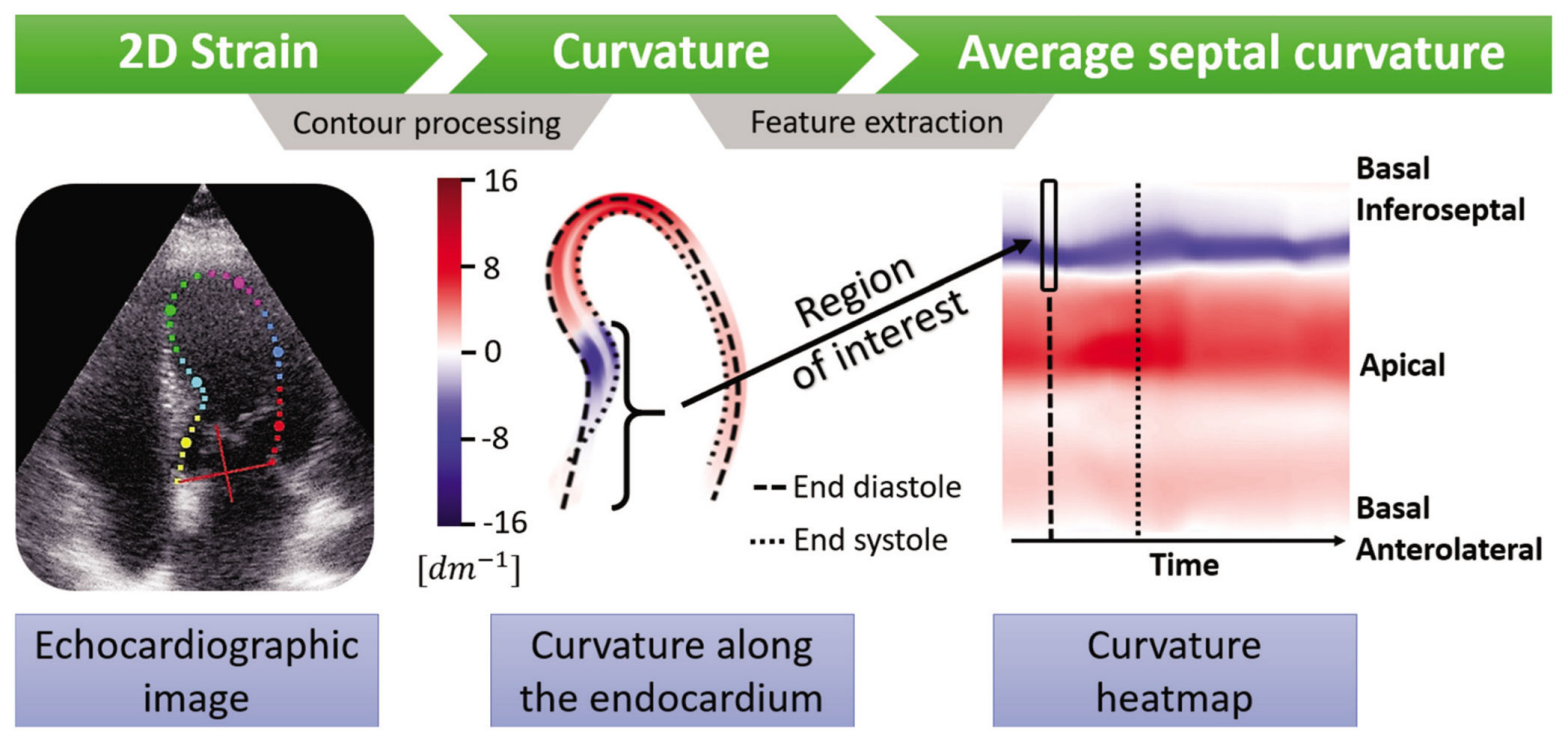
A proposed pipeline to go from ultrasound images to curvature analysis. The images from a full cardiac cycle are semi-automatically segmented with a 2D strain tool. Then, the contours of the endocardium are extracted, smoothed and interpolated to create a continuous contour from which the curvature is calculated. Finally, the curvature indexes within the region of interest (30% of the contour) in end-diastole are aggregated to compute the average septal curvature.

The curvature indexes are calculated for all contour points using a semi-automatic pipeline (see Fig. 2). In a 4CH view, BSH is located within the basal and sometimes mid inferoseptal segments. In order to create a useful metric, the calculated curvature indices from these two segments are aggregated into a single average value, which we named the average septal curvature (ASC). We hypothesize that the ASC of patients with BSH is far more concave in these segments than among hypertensive patients without BSH (conf. Fig. 1b).

In order to measure the curvature of the septal wall, the two-dimensional (2D) LV endocardial contour of the end-diastolic frame (used in the diagnosis of BSH [14]) is required. In an attempt to maximize clinical translation, we use a widely available semi-automatic segmentation tool (EchoPAC, GE Ultrasound), designed to measure the longitudinal 2D strain and re-purposed in this work to extract LV endocardial contours through the entire cardiac cycle. A similar pipeline could be applied to other modalities, such as cardiac MRI or computed tomography.

Using GE EchoPAC strain module, the operator sets a number of points on the endocardium of the LV in the end-systolic phase. Then, the algorithm interpolates the given set, to create additional points, which resemble a smooth curve. The operator then adjusts the segmentation shape by re-positioning the original points. In the next step, the contours are propagated thorough the entire cardiac cycle with the speckle-tracking algorithm.

The contours are exported from the module for post-processing as further interpolation is required to avoid underestimating the curvature in critical points. A radial basis function interpolator is chosen, with the smoothness parameter set to the original number of points in the trace as this algorithm provides smooth first and second spatial derivative of the interpolated contour (required in Eq. 1). As a result, each contour is up-sampled to 500 points, a resolution that was empirically tested to lead to robust and smooth curvature values (details on the implementation can be found in [19]). The end-diastolic frame is found based on the maximum area enclosed by the contour. The curvature is calculated along the entire contour in that frame. Finally, the 30% of the curvature indices from the top of the septal base through basal and mid segments are aggregated to compute the ASC.

To test the variability of measurements, an inter-observer/intra-observer analysis was conducted on a group of 20 patients: 10 healthy controls and 10 hypertensive patients, including two cases with BSH. The measurements were performed by clinical experts. The metrics were compared on the basis of the average absolute difference between the measurements relative to the range of measurements, to account for different metric units.

First, to evaluate intra-observer variability, the original observer (O1) remeasured the basal and mid-wall IVS dimensions in end-diastole and performed segmentation on all 20 patients at least 2 months after the original measurement (O1*). Second, to evaluate inter-observer variability, a different observer (O2) measured the same patients using the same 4CH and PLAX images that O1 used. Finally, another observer (O3) measured the same patients using a different 4CH image from the one used by O1 and O2. For most patients, only a single high-quality PLAX image was available, so the IVS was measured in the same PLAX image by O3 as O2 and O1. Similarly, the semiautomatic 4CH segmentation was performed by two other observers on the same (O2) and different (O3) images. This last study gives a measure of inter-observer inter-image variability on 4CH images, which is an important measure of robustness as an ideal method should give consistent results across any image from a patient given reasonable image quality.

To capture the deterioration caused by hypertension, functional metrics proven to be affected by BSH [7] were studied, including local longitudinal LV septal strain, longitudinal conduit and contractile left atrial strain (as well as ratio of the two), diastolic function marker (E/A), and mitral annulus septal and lateral velocities. Moreover, the metrics were studied against the anatomical markers of BSH, namely LV mass, and end-diastolic and end-systolic volumes indexed to BSA. The ability of both metrics, thickness and curvature, to infer the functional impairment is assessed by the regression between the variables within the hypertensive group (*N =* 161). The agreement between WTR (measured in two views) and ASC to identify BSH among the hypertensive population is studied by their correlation.

The processing pipeline, the statistical analysis and the variability analysis are implemented using the Python programming language, v.3.6.5. To test for normality of the distribution, the Pearson-D’Agostino test is used [20]. Two sample t-test is employed to test hypotheses on normally distributed samples, otherwise the Mann-Whitney *U* test is used. To calculate the correlation, Spearman’s p and Pearson’s *Rs* are computed.

## Results

**Figure 3:**
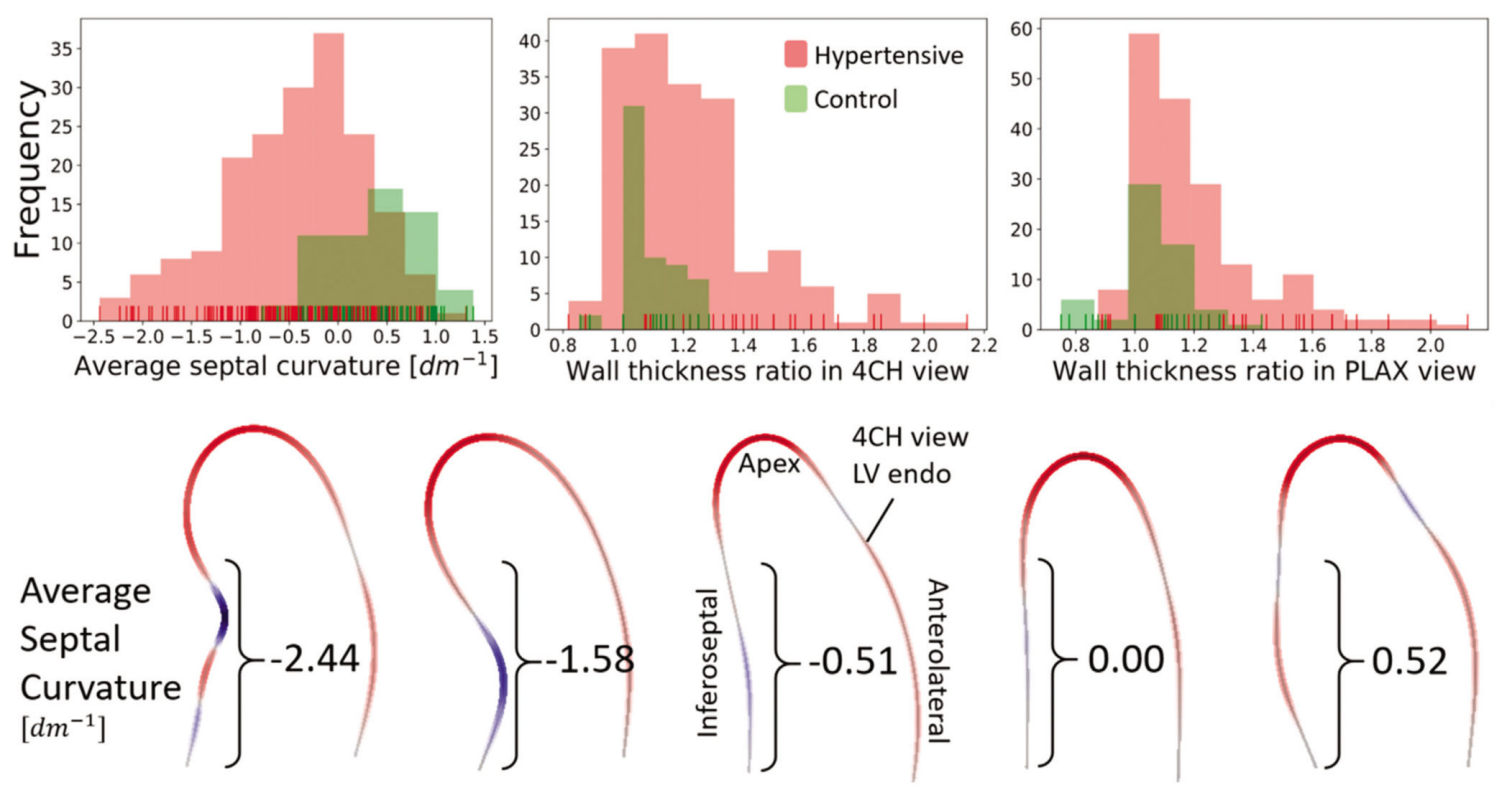
Upper: histograms of values of average septal curvature and wall thickness ratios measured in parasternal long-axis and four-chamber views among patients with and without hypertension. The negative values of the curvature represent concave curvature. The distribution of the curvature index resembles a normal distribution, whereas the wall thickness ratios are strongly skewed. Lower: 4CH view endocardial contours and their corresponding ASC values. The metric penalizes the sharp changes in the septal profile, regardless of its position. The two leftmost cases were diagnosed with BSH. 4CH, four-chamber; ASC, average septal curvature; BSH, basal septal hypertrophy.

As ASC is a novel metric, the distribution analysis is provided as a reference. The distributions of the three metrics under study, the ASC and WTR in 4CH and PLAX, in both the 161 hypertensive and 59 normotensive patients are depicted in Fig. 3. Moreover, the relevant percentiles and distribution information is shown in Table 1. The hypertensive population displays with a sharp transition around the ratio of 1.4 in a WTR in 4CH view, in concordance with the threshold for diagnosis of BSH. This transition is less clear in the WTR of the PLAX view, and no separation can be seen in the ASC distribution. None of the hypertensive distributions are normal (conf. Table 1). ASC is negatively skewed, with median value equal to concave curvature of −0.362 dm^−1^, and 75% of the cases have ASC below −0.88 dm^−1^. Conversely, WTR distributions are positively skewed, and the 85th percentile denotes the threshold for the diagnosis of BSH with respect to the WTR in either view.

In contrast to the hypertensive population, the distribution of the curvature among the healthy controls was mainly convex (median = 0.44dm^-1^, 78% cases with convex curvature index). This agrees with the understanding that the concave (‘sigmoid’) septum is a sign of a specific pattern of remodelling [14,21]. Representative examples with the ASC values are provided in Fig. 3.

The intra-observer and inter-observer differences relative to the range of measurements were significantly lower for the curvature index when compared with WTR (conf. Table 2). In fact, the two cases originally identified as HTN w/BSH were mismatched with the control cases when compared among observers using WTR. There was no systematic bias within and between observers in ASC and WTR_PLAX_ measurements. However, in the metric obtained from 4CH view, there was a significant interobserver discrepancy, where WTR measurements were on average 0.28 and 0.2 higher than those obtained by O2 and O3, respectively. The measurements and Bland-Altman plots are provided in the supplementary material (Figure S1, Tables S1-S3, http://links.lww.com/HJH/B583).

**Figure 4:**
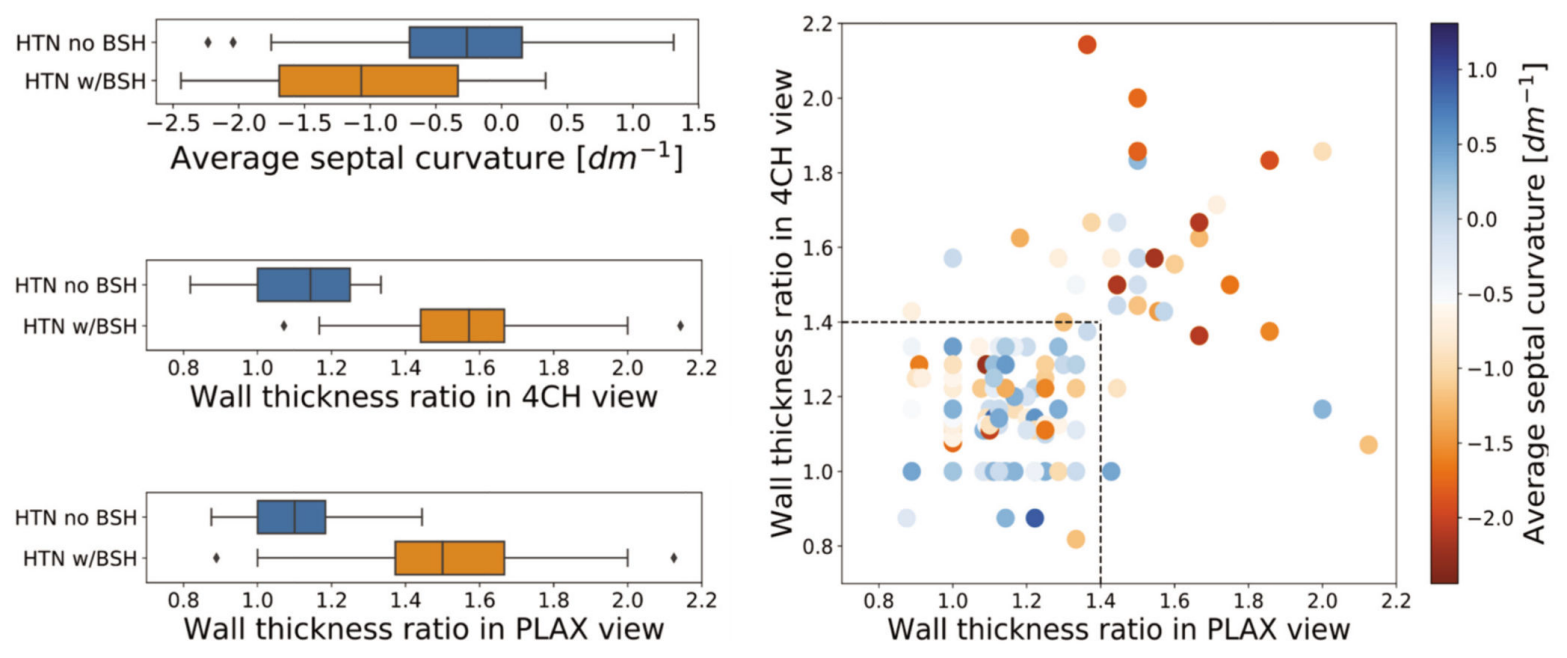
Left: box plots of values of average septal curvature and wall thickness ratios measured in parasternal long-axis and four-chamber views among patient: diagnosed and not diagnosed with BSH. Right: WTR values measured in two ultrasound views: PLAX and 4CH. The colours signify the values of ASC. The discrepancies between two metrics increase with the calculated values. The majority of the cases with WTRs below the BSH threshold also hold a low ASC value. 4CH, four-chamber; ASC, average septal curvature; BSH, basal septal hypertrophy.

Assuming current clinical standard the ground truth to identify the presence of BSH in hypertensive patients, we studied how each of the three individual metrics agreed with it. Given that WTR was used as the traditional metric to diagnose BSH, it was not surprising to find a better separation between groups with the WTR indexes than with ASC (left panel in Fig. 4). Nevertheless, the difference between the two groups was significant in all metrics (*P* < 0.001 in all cases).

A more detailed inspection of the metrics in a scatter plot (see right panel in Fig. 4) revealed the disagreements of the WTR acquired in different views. These discrepancies were more pronounced in the higher end of the WTR values, above the threshold value. The correlation between the two measurements was moderate (R = 0.36, P < 0.001). ASC was found to also be moderately correlated with the WTR in 4CH view (R =-0.4, P<0.001) and weakly correlated to the WTR in PLAX view (*R* =-0.21, *P =* 0.004). Stronger correlation between WTR_4CH_ and ASC measurements was expected as the curvature indexes were calculated from trace delineated in the 4CH view.

**Figure 5:**
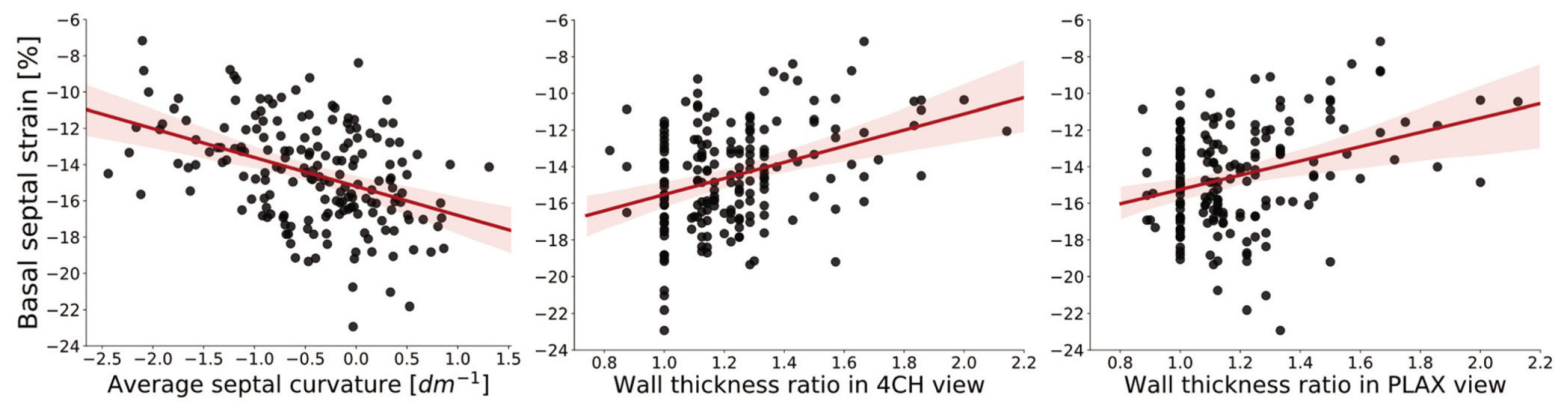
Linear regression between functional (basal septal strain index) and anatomical markers of basal septal hypertrophy: average septal curvature and wall thickness ratio indexes.

Although the new method does not classify the cases in the same manner, the relation between the metrics and cardiac deformation revealed that ASC was more correlated with the functional remodelling than with the anatomical markers: Table 3 shows that whenever the markers are significantly correlated with a functional parameter, the correlation with ASC is the strongest, except for medial *a’*. ASC is stronger correlated with all the strains, both ventricular and atrial, and the *E/A* ratio, with the most apparent difference in the basal longitudinal strain (shown in Fig. 5, *p*_ASC_ = -0.417 vs. *p*_4CH_ = 0.341 and *p*_PLAX_ = 0.24), which is also the most pronounced marker of BSH. In case of the mid-septal strain and left atrial conduit strain, ASC is the only significantly correlated metric. The function of mitral annulus in lateral location is not significantly correlated with any marker.

## Discussion

Average curvature of the septal segment is proposed as an anatomical feature to identify the presence of BSH. It is a more precisely defined and reproducible metric than thickness ratios, and better infers the functional remodelling of the basal septal segment.

The problem addressed in this work is the high variability between multiple WTR measurements, as described in the literature [22,23] and confirmed in our inter-observer and intra-observer analysis that indicated that 2D measurements of the septum thicknesses are not consistent (13 and 38% for intra-observer and inter-observer variability, see Table 2). This large variability led to discrepancies in diagnosis among the observers, making multiple control cases considered as having BSH and vice versa. The proposed solution is a quantitative definition of the so far qualitative visual assessment of the presence of the sigmoid septum currently done in clinical practice. ASC indices are more user-independent (7 and 8% for intra-observer and inter-observer variability, Table 2) and show better diagnosis agreement across observers.

ASC is more reproducible than WTR for two reasons. First, as it has a precise definition, on the contrary to WTR where the locations of the minimum and maximum thickness measurements are ambiguous. ASC is computed along the basal and mid inferoseptal segments of the left ventricle in a 4CH view, objectively derived as 30% of the LV endocardial contour. The second reason is that ASC only requires segmentation on the bright LV endocardial border: curvature defined on the LV endocardial border removes the source of variability in the delineation of the epicardium, which is usually more challenging and required in WTR.

There are still two potential sources of variability in ASC. First, there could be inconsistency in placing the first basal points of the endocardial contour, but its impact should be small because of its rigorous description in guidelines [17]. Second, the variability during image acquisition in positioning the basal segment in face of the outflow tract is an unsolved problem [24]. As an example, a few outliers exhibiting strong concave curvature among the non-BSH patients (conf. Fig. 4) were caused by low acquisition quality, where the 4CH view image almost entirely captured the left ventricular outflow tract and was thus closer to an apical long-axis view. The segmentation in the presence of the outflow track (e.g. on the apical long-axis view) lacks the consensus on the reproducible border between the ventricle and outflow, that would allow for the usage of curvature in this context. Despite these two potential sources of variability, ASC has shown to lead to a reproducible and easily interpretable metric, and could be further refined for other echocardiographic views, as well as other imaging modalities and 3D images. In addition, curvature could be defined in other echo views, such as the PLAX, provided that the guidelines were established.

Beyond reproducibility, and in the absence of outcome metrics and disease onset, this work searched for the BSH metric that correlated with those functional markers that indicate early signs of degradation. Changes in left atrial and ventricular function have been shown to be present in hypertensive patients with BSH – decreased regional LV systolic contraction was related with impaired LV relaxation, a higher level of indeterminate diastolic dysfunction and LA functional impairment [7]. Therefore, BSH has been linked to both morphological and functional cardiac remodelling, potentially increasing the patient’s risk for atrial fibrillation and heart failure. In our study, ASC shows a better ability to infer function impairment, and with the additional strength of being independent from LV mass and volume indexes making it a complementary anatomical marker, as opposed to the existing WTR markers (conf. Table 3). In addition, ASC is a metric that changes in patients with hypertension and is strongly amplified in BSH patients (conf. Fig. 3), suggesting its potential value in tracking the adaptation of the heart to the hypertensive insult. Further research is needed to investigate the ASC utility in risk quantification and prediction of disease onset or clinical outcomes. In detail, a longitudinal study showcasing the speed and pattern of local remodelling and changing curvature would shed light on the BSH development. In addition, ASC in BSH cases should be compared with other diseases related to thickening of the septal segments, such as hypertrophic cardiomyopathy.

Previous studies have explained the mechanistic cause of BSH by the extra stress that this region holds compared with the rest of the left ventricle when pressure increases [2,3,5]. Given a certain pressure in a chamber, the stress that a wall holds depends on its curvature accordingly to Laplace’s law: the flatter the surface, the larger the stress. The septum near the outflow track is the flattest anatomical part of the left ventricle, and thus becomes the early beacon of the presence of cardiac remodelling caused by hypertension. Our study of curvature provides additional evidence for this mechanistic explanation: the curvature in the septal region is on average flat (ASC = 0), or even convex (positive ASC), in many control patients (see Fig. 3). One interesting hypothesis is the existence of morphological features in the septum and outflow track that make the LV more prone to the occurrence of BSH: patients that have null or negative ASC values at the onset of the hypertension would be more sensitive to BSH in the progression of the disease.

The future methodological research will focus on the automation of the metric acquisition and unveiling the remodelling patterns, to classify the adaptive and maladaptive responses. It has recently been shown that deep neural networks can accurately segment the left ventricle, myocardium and the left atrium directly from ultrasound B-mode images [25]. Employing the networks could speed up generating the contours and create an objective metric independent of the observer, provided that the network could accurately delineate the septal profile. Furthermore, the statistical shape analysis has shown promise in aiding diagnosis and risk stratification [26,27]. This type of analysis could be directly applied to the segmented LV, to provide *z* scores describing the progress of the BSH in hypertensive patients. With such models and follow-up data, both maladaptive (increasing afterload) and adaptive (response to treatment) remodelling predictions could be made, to ensure appropriate treatment.

In conclusion, a novel shape biomarker, the LV endocardial septal curvature, was proposed to detect and quantify the presence of BSH. The distribution of this biomarker in control and hypertensive cohorts was described. It was found to be a more reproducible metric than thickness ratios and it better infers the functional deterioration related to hypertension. The tool to compute it [19], and the data to verify its implementation [28], are released to enable an easy adoption by the community.

## Supplementary Material

Supplementary material

## Figures and Tables

**Table 1:** The description of the distributions of average septal curvature and wall thickness ratio indexes

		Wall thickness ratio	
Distribution description	Average septal curvature (dm ^−1^)	4CH	PLAX
Minimum	−2.440	0.818	0.875
*P*_15_	−1.163	1	1
*P*_25_	−0.88	1.091	1
*P*_50_	−0.362	1.167	1.125
P_75_	0.041	1.333	1.286
*P*_85_	0.335	1.429	1.413
Maximum	1.310	2.143	2.125
*P*_normal_	0.035	<0.001	<0.001

*P*. values denote the percentiles. *P*_85_ of WTR in either of the views is the threshold for the diagnosis of BSH. *P*_normal_ is the *P* value of the test for normality. None of the distributions can be qualified as normal. 4CH, four-chamber; PLAX, parasternal long-axis.

**Table 2:** Results of intra-observer and inter-observer variability

			Absolute values			Relative values		
Obsever	Metric	Range	Max AD	AAD	SDAD	Max AD	AAD	SDAD
O1 and O1*	ASC	2.78	0.64	0.17	0.18	**23%**	**6%**	**7%**
	WTR_4CH_	0.88	0.52	0.11	0.12	59%	12%	13%
	WTR_PLAX_	0.94	0.45	0.16	0.12	47%	17%	13%
O1 and O2	ASC	2.85	0.67	0.23	0.20	**23%**	**8%**	**7%**
	WTR_4CH_	0.86	0.77	0.28	0.22	89%	33%	25%
	WTR_PLAX_	0.89	0.56	0.26	0.14	63%	30%	16%
O1 and O3	ASC	2.39	0.74	0.32	0.22	**31%**	**13%**	**9%**
	WTR_4CH_	0.51	0.98	0.26	0.26	190%	51%	51%
	WTR_PLAX_	0.67	0.67	0.18	0.17	100%	26%	26%

Differences in absolute and relative (to the range of the index) terms. Bold indicates most relevant values. 4CH, four-chamber; AD, absolute difference; ASC, average septal curvature; AAD, average AD; BSH, basal septal hypertrophy; SDAD, standard deviation of AD.

**Table 3:** Rank-correlation between average septal curvature and wall thickness ratios markers and anatomical and functional parameters

	Spearman *ρ*		
Variables	ASC	WTR_4CH_	WTR_PLAX_
LV mass index	0.018	0.162*	0.173*
LV end-diastolic volume index	0.062	0.079	0.157*
LV end-systolic volume index	0.113	0.060	0.122
2D left atrial conduit volume	0.051	0.057	0.100
*E/A* ratio	0.187*	−0.163*	−0.131
Mitral annulus e′ medial velocity	0.234*	−0.156*	−0.158*
Mitral annulus a′ medial velocity	−0.192*	0.227*	0.163*
Mitral annulus e′ lateral velocity	0.062	−0.116	−0.085
Average mitral annulus e’ velocity	0.129	0.148	0.137
Basal septal strain	−0.417*	0.341*	0.24*
Mid septal strain	−0.164*	0.100	0.109
LA contractile strain	−0.219*	0.158*	0.152
LA conduit strain	0.159*	−0.137	−0.060
LA conduit/contractile strain ratio	−0.232*	0.203*	0.140

ASC, average septal curvature; BSH, basal septal hypertrophy; LA, left atrium; LV, left ventricle; WTR, wall thickness ratio.

* *P* value less than 0.05 for correlation.

## Abbreviations

2D: two-dimensional
ASC: average septal curvature
BSH: basal septal hypertrophy
IVS: interventricular septum
WTR: wall thickness ratio
