# Supplementary material for: Septal curvature as a robust and reproducible marker for basal septal hypertrophy
Source: J Hypertens. Author manuscript; Available in PMC 2021 Jul 1. (PMC8183485; doi:10.1097/HJH.0000000000002813)
Supplement: Supplementary material [file EMS127202-supplement-Supplementary_material.docx]

Septal Curvature as a Robust and Reproducible Marker for Basal Septal Hypertrophy

**Supplementary material**

Maciej MARCINIAK^1^*, MSc. Eng., Andrew GILBERT^2^, MSc. Eng., Filip LONCARIC^3^, MD, Joao Filipe FERNANDES^1^, MSc. Eng., Bart BIJNENS^34^, MSc. Eng. PhD, Marta SITGES^5^, MD, PhD

Andrew KING^1^, MSc. Eng. PhD, Fatima CRISPI^356^, MD, PhD, Pablo LAMATA^1^, MSc. Eng. PhD

**Institutions:**

1 - School of Biomedical Engineering and Imaging Sciences, Kings College London, London, United Kingdom

2 - Cardiovascular Ultrasound, GE Vingmed, Oslo, Norway

3 - Institute of Biomedical Research August Pi Sunyer (IDIBAPS), Barcelona, Spain

4 - Catalan Institution for Research and Advanced Studies (ICREA), Barcelona, Spain

5 - Hospital Clinic de Barcelona, Barcelona, Spain

6 - Barcelona Center for Maternal Fetal and Neonatal Medicine, Hospital Sant Joan de Déu, Barcelona, Spain

Supplementary material consists of Table S1 with demographics of the study participants, Tables S2-4 with the wall thickness ratios and average septal curvature measurements and variability among the observers and Figure S1 with the Bland-Altman plots for all metrics and the three observers.

**Table S1:** **Patient demographics**.

| **Biometric** | **Healthy controls** | **Basal Septal Hypertrophy** | | **p-value** |
| --- | --- | --- | --- | --- |
|  | **(N=59)** | **No (N=129)** | **Yes (N=32)** |  |
| **Age [years]** | 36 (35-40) | 56 (40-66)* | 58 (42-66)*# | #0.03 |
| **Men** | 32 (55%) | 70 (48%) | 12 (33%) |  |
| **Body Surface**  **Area [m2]** | 1.81 ± 0.22 | 1.91 ± 0.23* | 1.98 ± 0.20* | 0.10 |
| **Body Mass**  **Index [kg/m2]** | 25.1 ± 4.3 | 28.0 ± 4.5* | 28.4 ± 3.9* | 0.38 |
| **Heart Rate**  **[bpm]** | 72 ± 11 | 67 ± 10* | 69 ± 10* | 0.60 |
| **Systolic blood pressure [mmHg]** | 117 ± 12 | 136 ± 15* | 141 ± 14*# | #0.04 |
| **Diastolic blood pressure [mmHg]** | 72 ± 7 | 80 ± 10* | 83 ± 10* | 0.11 |

* - Significant difference between the group and the control group.

# - Significant difference between the hypertensive groups.

**Table S2: Average septal curvature measurements and corresponding intra and inter-observer variability.**

| **Average Septal Curvature** | | | | | | | **Inter- and intra-observer variability**  **Difference** | | |
| --- | --- | --- | --- | --- | --- | --- | --- | --- | --- |
| **O1** | | **O1*** | | **O2** | | **O3** | **O1 & O1*** | **O1 & O2** | **O1 & O3** |
| -0.07 | -0.10 | | 0.08 | | -0.01 | | 0.03 | -0.14 | -0.06 |
| 0.34 | 0.80 | | 0.36 | | 0.45 | | -0.46 | -0.02 | -0.11 |
| 0.60 | 0.66 | | 0.71 | | 0.19 | | -0.06 | -0.11 | 0.42 |
| 0.54 | 0.46 | | 0.20 | | 0.45 | | 0.08 | 0.34 | 0.09 |
| -0.28 | -0.30 | | -0.50 | | -0.20 | | 0.02 | 0.22 | -0.07 |
| 0.07 | 0.36 | | -0.32 | | -0.50 | | -0.29 | 0.38 | 0.56 |
| -0.14 | 0.09 | | -0.14 | | 0.16 | | -0.23 | 0.00 | -0.30 |
| -0.41 | 0.17 | | -0.25 | | 0.16 | | -0.58 | -0.16 | -0.57 |
| -0.16 | -0.08 | | 0.30 | | 0.32 | | -0.09 | -0.46 | -0.48 |
| -0.64 | -0.56 | | -0.65 | | -0.12 | | -0.08 | 0.01 | -0.53 |
| -0.77 | -0.94 | | -0.94 | | -1.21 | | 0.17 | 0.17 | 0.45 |
| -0.81 | -0.74 | | -0.14 | | -1.18 | | -0.07 | -0.67 | 0.37 |
| -2.11 | -2.18 | | -2.28 | | -1.68 | | 0.07 | 0.17 | -0.43 |
| -0.11 | -0.20 | | -0.29 | | -0.06 | | 0.09 | 0.18 | -0.05 |
| -0.44 | -0.56 | | -0.34 | | -0.46 | | 0.12 | -0.10 | 0.02 |
| -1.65 | -0.99 | | -0.98 | | -1.04 | | -0.66 | -0.67 | -0.61 |
| -0.80 | -0.78 | | -1.03 | | -0.87 | | -0.02 | 0.24 | 0.08 |
| 0.33 | 0.32 | | -0.19 | | -0.43 | | 0.01 | 0.52 | 0.76 |
| -1.96 | -2.15 | | -1.88 | | -1.79 | | 0.19 | -0.08 | -0.17 |
| 0.01 | -0.03 | | 0.02 | | 0.31 | | 0.04 | -0.02 | -0.30 |
|  |  | |  | | **Average** | | -0.08 | -0.01 | -0.05 |
|  |  | |  | | **SD** | | 0.23 | 0.31 | 0.39 |

**Table S3: Wall thickness ratio measurements performed in 4CH view and corresponding intra and inter-observer variability.**

| **4CH view IVS thickness ratio between basal and mid segments** | | | | | | | **Inter- and intra-observer variability**  **Difference** | | |
| --- | --- | --- | --- | --- | --- | --- | --- | --- | --- |
| **O1** | | **O1*** | | **O2** | | **O3** | **O1 & O1*** | **O1 & O2** | **O1 & O3** |
| 1.00 | 1.17 | | 0.67 | | 1.00 | | -0.17 | 0.33 | 0.00 |
| 1.14 | 1.00 | | 0.67 | | 0.88 | | 0.14 | 0.48 | 0.27 |
| 1.13 | 1.00 | | 0.67 | | 1.14 | | 0.13 | 0.46 | -0.02 |
| 1.00 | 1.00 | | 0.88 | | 1.17 | | 0.00 | 0.13 | -0.17 |
| 1.00 | 1.14 | | 0.88 | | 1.14 | | -0.14 | 0.13 | -0.14 |
| 1.10 | 1.22 | | 0.89 | | 1.00 | | -0.12 | 0.21 | 0.10 |
| 1.71 | 1.75 | | 1.00 | | 0.90 | | -0.04 | 0.71 | 0.81 |
| 1.14 | 1.17 | | 1.00 | | 1.00 | | -0.02 | 0.14 | 0.14 |
| 1.00 | 1.00 | | 1.00 | | 1.14 | | 0.00 | 0.00 | -0.14 |
| 1.11 | 1.11 | | 0.86 | | 0.91 | | 0.00 | 0.25 | 0.20 |
| 1.00 | 1.11 | | 0.86 | | 0.89 | | -0.11 | 0.14 | 0.11 |
| 1.13 | 1.00 | | 0.83 | | 0.88 | | 0.13 | 0.29 | 0.25 |
| 1.63 | 2.14 | | 0.86 | | 1.00 | | -0.52 | 0.77 | 0.63 |
| 1.00 | 1.00 | | 0.86 | | 0.75 | | 0.00 | 0.14 | 0.25 |
| 1.22 | 1.13 | | 0.92 | | 0.70 | | 0.10 | 0.31 | 0.52 |
| 1.11 | 1.29 | | 0.55 | | 0.78 | | -0.17 | 0.57 | 0.33 |
| 1.11 | 1.13 | | 1.13 | | 1.17 | | -0.01 | -0.01 | -0.06 |
| 1.00 | 1.00 | | 0.86 | | 1.00 | | 0.00 | 0.14 | 0.00 |
| 1.88 | 1.67 | | 1.50 | | 0.90 | | 0.21 | 0.38 | 0.98 |
| 1.00 | 1.17 | | 1.00 | | 1.14 | | -0.17 | 0.00 | -0.14 |
|  |  | |  | | **Average** | | -0.04 | 0.28 | 0.20 |
|  |  | |  | | **SD** | | 0.15 | 0.22 | 0.32 |

**Table S4: Wall thickness ratio measurements performed in PLAX view and corresponding intra and inter-observer variability.**

| **PLAX view IVS thickness ratio between basal and mid segments** | | | | | | | **Inter- and intra-observer variability**  **Difference** | | |
| --- | --- | --- | --- | --- | --- | --- | --- | --- | --- |
| **O1** | | **O1*** | | **O2** | | **O3** | **O1 & O1*** | **O1 & O2** | **O1 & O3** |
| 1.33 1.00 1.33 0.75  1.00 0.90 1.29 0.70  0.90 1.13 1.11 1.01  1.17 1.00 0.88 1.14  0.67 0.78 1.00 0.78  1.57 1.25 1.83 0.68  1.38 1.71 1.86 0.92  1.17 1.00 1.50 0.67  1.00 1.00 1.43 0.70  1.00 1.11 0.80 1.39  1.43 1.25 1.20 1.04  1.25 1.11 0.89 1.25  1.36 1.36 0.80 1.70  1.00 1.00 0.63 1.60  1.57 1.13 1.67 0.68  1.25 1.00 1.20 0.83  1.13 1.00 1.00 1.00  1.00 1.13 1.14 0.98  1.67 1.67 1.44 1.15  1.13 1.00 1.43 0.701.00 | 1.17 | | 0.67 | | 1.00 | | 0.33 | 0.00 | 0.58 |
| 1.14 | 1.00 | | 0.67 | | 0.88 | | 0.10 | -0.29 | 0.30 |
| 1.13 | 1.00 | | 0.67 | | 1.14 | | -0.23 | -0.21 | -0.11 |
| 1.00 | 1.00 | | 0.88 | | 1.17 | | 0.17 | 0.29 | 0.02 |
| 1.00 | 1.14 | | 0.88 | | 1.14 | | -0.11 | -0.33 | -0.11 |
| 1.10 | 1.22 | | 0.89 | | 1.00 | | 0.32 | -0.26 | 0.89 |
| 1.71 | 1.75 | | 1.00 | | 0.90 | | -0.34 | -0.48 | 0.45 |
| 1.14 | 1.17 | | 1.00 | | 1.00 | | 0.17 | -0.33 | 0.50 |
| 1.00 | 1.00 | | 1.00 | | 1.14 | | 0.00 | -0.43 | 0.30 |
| 1.11 | 1.11 | | 0.86 | | 0.91 | | -0.11 | 0.20 | -0.39 |
| 1.00 | 1.11 | | 0.86 | | 0.89 | | 0.18 | 0.23 | 0.39 |
| 1.13 | 1.00 | | 0.83 | | 0.88 | | 0.14 | 0.36 | 0.00 |
| 1.63 | 2.14 | | 0.86 | | 1.00 | | 0.00 | 0.56 | -0.34 |
| 1.00 | 1.00 | | 0.86 | | 0.75 | | 0.00 | 0.38 | -0.60 |
| 1.22 | 1.13 | | 0.92 | | 0.70 | | 0.45 | -0.10 | 0.90 |
| 1.11 | 1.29 | | 0.55 | | 0.78 | | 0.25 | 0.05 | 0.42 |
| 1.11 | 1.13 | | 1.13 | | 1.17 | | 0.13 | 0.13 | 0.13 |
| 1.00 | 1.00 | | 0.86 | | 1.00 | | -0.13 | -0.14 | 0.02 |
| 1.88 | 1.67 | | 1.50 | | 0.90 | | 0.00 | 0.22 | 0.51 |
| 1.00 | 1.17 | | 1.00 | | 1.14 | | 0.13 | -0.30 | 0.43 |
|  |  | |  | | **Average** | | 0.07 | -0.02 | 0.21 |
|  |  | |  | | **SD** | | 0.19 | 0.30 | 0.39 |

**Figure S1: Bland-Altman plots reporting on the intra- and inter-observer reproducibility study in 20 subjects.**


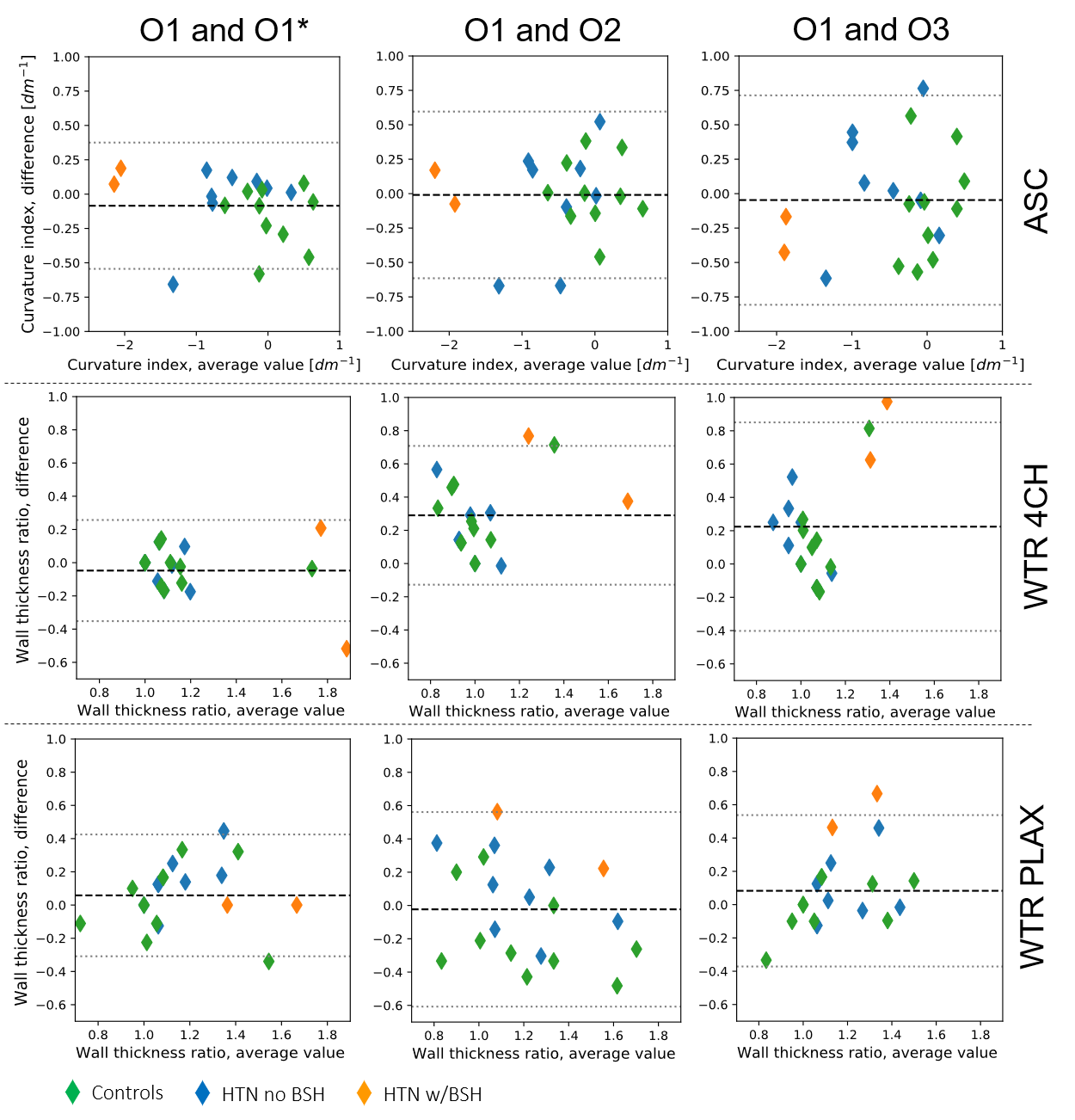


The cases diagnosed with BSH retained a strongly concave ASC among all observers. In case of WTR, there are strong discrepancies, where the controls may be considered as having BSH, different number of cases can be diagnosed with BSH depending on the observers, and a strong bias can be found in the measurements performed in 4CH view.

*[Coloured]*
